# Ileocecal Intussusception with Histomorphological Features of Inflammatory Neuropathy in Adenovirus Infection

**DOI:** 10.1155/2009/579501

**Published:** 2010-02-11

**Authors:** Elke Kaemmerer, Jens J. W. Tischendorf, Gerd Steinau, Norbert Wagner, Nikolaus Gassler

**Affiliations:** ^1^Department of Pediatrics, RWTH Aachen University, 52074 Aachen, Germany; ^2^Department of Medicine III, RWTH Aachen University, 52074 Aachen, Germany; ^3^Department of Surgery, RWTH Aachen University, 52074 Aachen, Germany; ^4^Institute of Pathology, RWTH Aachen University, 52074 Aachen, Germany

## Abstract

The pathophysiological mechanisms for ileocecal intussusception in children with adenovirus infection are not well characterized. Here we demonstrate coincidence of adenovirus infection and inflammatory neuropathy of myenteric plexus in two children with ileocecal intussusception. Inflammatory neuropathy, an unspecific morphological feature which is found in peristalsis disorders, was morphologically characterized by the influx of CD3 positive lymphocytes in nervous plexus. To our knowledge, this is the first report suggesting peristalsis disorders from inflammatory neuropathy as additional mechanism in the pathophysiological concept of adenovirus-associated ileocecal intussusception.

## 1. Introduction

The classification of intestinal intussusception—the invagination of an intestinal segment into another—is generally based on anatomic considerations including enteric, ileocecal, colocolic, as well as rectoanal intussusception, and the subtypes ileocecocolic, ileoileocolic, and ileocolocecocolic intussusception [1].

Ileocecal intussusception is found with high frequency in infants and young children. The incidence is assumed with 0.66 to 2.24 per 1.000 children in inpatient departments and from 0.75 to 1.00 in emergency facilities with peak incidence in patients of 3–9 months of age [2]. The four classical symptoms include emesis, abdominal pain, a palpable abdominal mass/tumour, and rectal bleeding. There is high agreement that ultrasound is the most reliable diagnostic procedure in patients suspicious for intussusception, and conservative enema treatment is generally recommended [3].

Intussusception is of low frequency in adults when compared with infants and young children [4]. It is suggested that this discrepancy is based on structural and functional specialities of the intestine. From this point of view the variable disproportion between ileal and cecal segment diameters, ileal projection through the cecal wall during discharge of intestinal content, mobility of the ileocecal region as itself, and nervous maturation at the junction of the vagal and sacral portions of the parasympathetic system are proposed as relevant parameters [1].

Ileocecal intussusception is regarded idiopathic in the overwhelming number of patients because an underlying disorder is not detectable. However, some evidence is given that ileocecal intussusception in children might be associated with intestinal viral infections especially rota-, astro-, or adenovirus [1, 5–7]. It is suggested that susceptible individuals may have an altered immune and/or anatomic status predisposing to intussusception [7, 8]. In such patients pathophysiological relevance is attributed to the enlargement of Peyer's patches and/or mesenteric lymph nodes which could probably act as a leading edge [1, 5, 6, 9, 10]. Exuberant lymphoid hyperplasia is not generally found in surgical specimens of affected patients. Therefore it is hypothesized that additional mechanisms promoting intussusception could exist. At present, inflammatory neuropathy, an unspecific morphological feature of peristalsis disorders, is not well described in patients with adenovirus-associated intussusception. An immunohistochemical characterization of inflammatory infiltrates affecting intramural nervous plexus is not available.

Our data argue for CD3-triggered inflammatory neuropathy, which probably promotes peristalsis disorders, as additional mechanism in the pathogenesis of ileocecal intussusception in adenovirus infection.

## 2. Case Report 1: Ileocecal Intussusception and Adenovirus Infection

### 2.1. Clinic

A 4-year-old boy with diarrhoea and abdominal pain over some hours, but without emesis or rectal bleeding, was admitted to the University hospital. Except for bronchitis years ago, anamnestic data were unremarkable. Manual investigation did not reveal an abdominal mass, but in auscultation peristalsis was diminished. Abdominal sonography revealed features of ileocecal intussusception. Testing for adenovirus from stool probes was positive. In order to disintegrate intussusception, enemas were performed controlled with radioscopy. This imaging technique displayed a bilobulated mucosa-associated mass in the terminal ileum. Since all attempts to dissolve intussusception were not successful, surgical intervention and ileocecal resection were performed. Surgical intervention, postoperative phase, and two-year follow-up were without any complication.

### 2.2. Macroscopy

**Figure 1:**
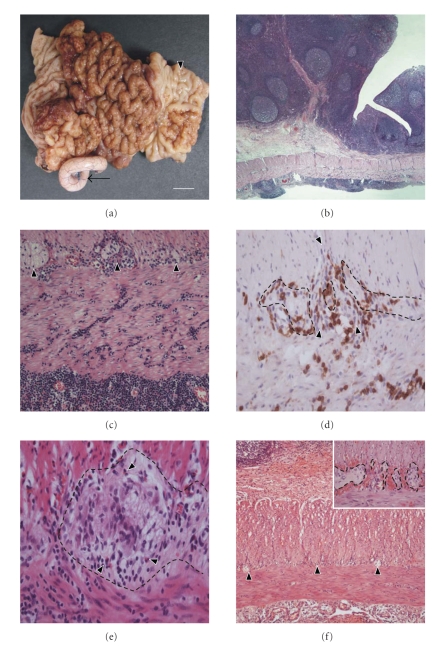
Morphological aspects of ileocecal surgical specimens with intussusception; (a)–(d) Patient 1 (adenovirus infection); (e) Patient 2 (adenovirus infection); (f) Patient 3 (no adenovirus infection). (a) Ileocecal specimen with polypoid intraluminal tumour nodules (arrowhead). The appendix is marked by an arrow. Barr indicates 1 cm. (b) Transmural tissue section of the terminal ileum displaying lymphoid hyperplasia with diffuse lymphocytic infiltration. H/E; original magnification 50×. (c) Dense lymphocytic infiltrates in strong vicinity to nervous plexus with ganglia (arrowheads). Lymphocytes accumulate in subserosal connective tissues. H/E; original magnification 200×. (d) Anti-CD3 immunohistochemistry demonstrates T-lymphocytes in strong vicinity to the intramural plexus cells (plexus border is marked by dotted line). Small vessels crossing the plexus are marked with arrowheads. Original magnification 400×. (e) Tissue section of the terminal ileum (patient 2) demonstrates the plexus myentericus (dotted line) and several infiltrating lymphocytes and eosinophiles (arrowheads). H/E; original magnification 400×. (f) Transmural ileal tissue section (patient 3, adenovirus negative intussusception) with only mild lymphoid hyperplasia and few infiltrating lymphocytes (the nervous plexus is marked by arrowheads). Inset: Strong accumulation of erythrocytes adjacent to the nervous plexus (dotted line), but morphological features of inflammatory neuropathy are not visible. H/E; original magnification 50×; inset 400×.

The surgical specimen included 4 cm ileal and 6 cm colon segments, 8 cm appendix, and 3 cm mesenteric fatty tissue. Adjacent to Bauhin's valve, ileal mucosa displayed a macro- and micronodular polypoid mass with a maximal diameter of approximately 8 cm (Figure 1(a)). The gyrated surface structure included some tissue erosions and morphological features of chronic tissue damage and bleeding. Diffuse thickening of the intestinal wall and micronodular transformation of serosa tissues were focally visible.

### 2.3. Microscopy

Histomorphological evaluation of the polypoid intraluminal mass revealed compact arranged monomorphous small lymphocytes frequently intermingled with enlarged follicles (Figure 1(b)). In follicle centres several macrophages containing condensed corpuscles in the cytoplasm were found. Lymph follicles were demarcated by a good visible mantle zone. In the lamina propria and other tissue layers, lymphatic and blood vessels were enlarged and extravascular erythrocytes were frequently seen. The surface lining epithelium was intermingled with neutrophiles and lymphocytes. Some epithelial microcrypts as well as focal depletion of goblet and Paneth cells were found. Although some apoptotic bodies were seen in the surface lining epithelium, intranuclear viral inclusions of the Cowdry types were not visible. In deeper layers of the ileal wall diffuse infiltrates of small-sized lymphocytes, lymphoid aggregates, and eosinophiles were found. Frequently, the infiltrating lymphocytes were in close contact to axons and ganglia of the enteric plexus and enriched in vascular channels (Figure 1(c)).

### 2.4. Immunohistochemistry

The lymphocytes displayed strong coexpression of CD3, CD8, and frequently Bcl2, whereas CD5 was only occasionally detectable (Figure 1(d)). A small number of deep penetrating lymphocytes were positive for CD20 and Bcl2, but the majority was negative for CD10 and CD23. In addition, macrophages (CD68) and mast cells (CD117) were diffusely arranged in the connective tissues. Immunostainings of S100 protein or GFAP did not reveal any feature of significant dislocation of plexus cells nor nerve cell degeneration.

## 3. Case Report 2: Ileocecal Intussusception and Adenovirus Infection

### 3.1. Clinic

A 3-year-old boy demonstrated clinical symptoms and adenovirus infection as detailed above. In contrast to patient 1, lymphoid hyperplasia was moderately established. Enema resistant ileocecal intussusception was the cause for surgical intervention. Surgical resection of the ileocecal segment and one-year follow-up were without any complication.

### 3.2. Macroscopy

The surgical specimen included a small and a large intestinal segment, each about 5 cm, a 6 cm appendix, and 3.5 cm mesenteric fatty tissue. In the formalin-fixed surgical specimen, ileocecal intussusception or a nodular mass was not found.

### 3.3. Microscopy and Immunohistochemistry

In tissue sections of the terminal ileum, lymphoid hyperplasia was only mild and the surface lining epithelium displayed only focal erosions. Intranuclear inclusion bodies indicating for adeno-virus infection were not detectable. The deeper tissue layers were strongly infiltrated by inflammatory cells including abundant CD3 positive lymphocytes. Features of inflammatory neuropathy with lymphocytes and eosinophiles in close contact to nervous cells of the plexus myentericus were found (Figure 1(e)).

## 4. Case Report 3: Ileocecal Intussusception without Adenovirus Infection

### 4.1. Clinic

A six-month-old girl demonstrated clinical symptoms of ileocecal intussusception and peritonitis. Stool tests for adenovirus or other infections were always negative. In abdominal sonography, lymphoid hyperplasia was mild. Surgical resection of the ileocecal segment was performed without complications. The half-year follow-up was unremarkable.

### 4.2. Macroscopy

The surgical specimen included a 13 cm small and a 6.5 cm large intestinal segment as well as the 5 cm appendix.

### 4.3. Microscopy and Immunohistochemistry

The histological findings were dominated by moderate lymphoid hyperplasia and strong erythrocyte extravasates. In the deep tissue layers, infiltrating lymphocytes were only few in number. Densely packed erythrocytes were found adjacent to the nervous plexus, but morphological hallmarks of inflammatory neuropathy, demonstrated in adenovirus-positive cases 1 and 2, were not visible (Figure 1(f)).

## 5. Discussion

Ileocecal intussusception is one of the most common surgical emergencies in children and a frequent cause of intestinal obstruction in infancy. There is wide agreement that ileocecal intussusception is strongly promoted by adenovirus infection and a leading edge which is preferentially formed by a tumour or a tumour-like lesion [6, 11].

Peyer's patches are a functional high dynamic lymphoid tissue of the terminal ileum which is responsive to several stimuli with formation of lymphofollicular hyperplasia. In children, intestinal viral infection with adenovirus, astrovirus, or rotavirus has been identified as important cause for exuberant lymphoid hyperplasia of Peyer's patches with development of a tumour-like mass. Some evidence is given that exuberant hyperplastic lymphoid tissues favour ileocecal intussusception as a leading edge [12]. It is known from literature [11] and demonstrated in this report (case 2) that in adenovirus infection establishment of ileocecal intussusception without exuberant hyperplasia of lymphoid tissues is possible. The percentage of an identifiable leading edge at the time of surgery is in large series below 10 percent [11]. In this scenario, additional pathophysiological mechanisms promoting the development of ileocecal intussusception in adenovirus infection are in discussion. To our knowledge, the attractive hypothesis, focal inflammatory neuropathy as adenovirus-related disorder with injury of local intestinal motility, is not addressed up-to-now.

In the present study, lymphocytic infiltration of nervous plexus, the morphological hallmark of inflammatory neuropathy, was found in two patients with adenovirus infection and intussusception (cases 1 and 2), but not in patient 3 (case 3: adenovirus negative intussusception). Our working hypothesis, that inflammatory neuropathy might be associated with injury of intestinal motility, is favoured by two recent studies by Lindberg and coworkers [13, 14]. In these studies, inflammatory neuropathy triggered by CD3 positive lymphocytes was characterized as a striking phenomenon and the underlying reason for enteric dysmotility and chronic intestinal pseudoobstruction. In this functional setting, inflammatory neuropathy could be assumed as a putative cofactor in the pathophysiological concept of adenovirus infection-associated intussusception.

Dense lymphocytic infiltration of intestinal plexus has mainly been reported in CMV or EBV infection, Chagas' disease, and as a paraneoplastic phenomenon [15, 16]. However, the patients presented here did not show any evidence for such disease.

Surgical manipulation of the intestine has been reported to cause infiltration of leukocytes (preferential neutrophiles) and macrophages into the bowel wall, but there are no reports demonstrating lymphocyte and eosinophile migration to nervous plexus. In our experience and illustrated in case 3, lymphocytes are not present in nervous plexus after extended surgical manipulation that contradict the idea that surgical intervention or enema might underlie our morphological finding of plexus inflammation.

The data demonstrated here argue for the attractive hypothesis of inflammatory neuropathy as putative variable in the pathophysiological concept of adenovirus-associated ileocecal intussusception in children.
